# The Effect of Visual Cues on Difficulty Ratings for Segregation of Musical Streams in Listeners with Impaired Hearing

**DOI:** 10.1371/journal.pone.0029327

**Published:** 2011-12-15

**Authors:** Hamish Innes-Brown, Jeremy Marozeau, Peter Blamey

**Affiliations:** 1 The Bionics Institute, Melbourne, Australia; 2 Department of Otolaryngology, University of Melbourne, Melbourne, Australia; McMaster University, Canada

## Abstract

**Background:**

Enjoyment of music is an important part of life that may be degraded for people with hearing impairments, especially those using cochlear implants. The ability to follow separate lines of melody is an important factor in music appreciation. This ability relies on effective auditory streaming, which is much reduced in people with hearing impairment, contributing to difficulties in music appreciation. The aim of this study was to assess whether visual cues could reduce the subjective difficulty of segregating a melody from interleaved background notes in normally hearing listeners, those using hearing aids, and those using cochlear implants.

**Methodology/Principal Findings:**

Normally hearing listeners (N = 20), hearing aid users (N = 10), and cochlear implant users (N = 11) were asked to rate the difficulty of segregating a repeating four-note melody from random interleaved distracter notes. The pitch of the background notes was gradually increased or decreased throughout blocks, providing a range of difficulty from easy (with a large pitch separation between melody and distracter) to impossible (with the melody and distracter completely overlapping). Visual cues were provided on half the blocks, and difficulty ratings for blocks with and without visual cues were compared between groups. Visual cues reduced the subjective difficulty of extracting the melody from the distracter notes for normally hearing listeners and cochlear implant users, but not hearing aid users.

**Conclusion/Significance:**

Simple visual cues may improve the ability of cochlear implant users to segregate lines of music, thus potentially increasing their enjoyment of music. More research is needed to determine what type of acoustic cues to encode visually in order to optimise the benefits they may provide.

## Introduction

Auditory sensation can often be partially restored in people with hearing impairment. When the impairment is mild to severe, amplification via a hearing aid (HA) can be sufficient. For the profoundly deaf, a cochlear implant (CI) is required. A CI is a neural prosthesis that directly stimulates the auditory nerve, bypassing missing or diseased cochlear hair cells. Cochlear implants are successful in restoring speech perception in most individuals [1], and hearing aids provide improvements to speech reception with hearing losses of up to 90 dB HL. While satisfaction ratings and performance of HAs are high in many listening situations [2], [3], the perception and appreciation of music, especially when using a CI, is far more problematic [4], [5]. Problems with the accurate perception of pitch play a large part in this issue, but accurate pitch perception is not the only factor in the enjoyment and appreciation of complex musical signals. The ability to perceive auditory ‘streams’ separately is also important in the appreciation of music.

Music often contains many such streams – for instance a melody and harmony played on a single instrument, or multiple melodies played by the same or separate instruments. Much of the skill of the composer is in manipulating the perception of these streams, and the ability to interpret them separately is vital to the appreciation of music. The capacity to separate and group auditory streams is called auditory stream segregation [6]. This ability is based on acoustic (or bottom-up) differences between streams, as well as cognitive (or top-down) factors, such as memory, expectation, experience, and information from other sensory sources [7]. One well-known acoustic cue that contributes to stream segregation is pitch. If low frequency (A) and high frequency (B) pure tones are played in a repeating ABA-ABA pattern, increasing the frequency difference (δF) between the A and B tones increases the likelihood that the pattern is perceived as segregated into A-A-A-A and -B---B- streams, rather than fused in a single ABA-ABA stream [8]. In addition to frequency, other perceptual differences between streams, such as localisation cues [9], [10], loudness [11], the temporal and spectral features contributing to timbre [7], or any other perceptual difference [12] can affect stream segregation. Unfortunately, the perception of many of these cues is degraded by sensorineural hearing loss, which reduces the perceptual differences between sound sources, in turn leading to a decreased ability to segregate auditory streams.

### How hearing devices affect streaming cues

A hearing aid processes and amplifies sound signals, and delivers acoustic stimulation to the eardrum via a small loudspeaker in or near the ear canal. The type of signal processing applied varies between devices and prescribed fittings, but can include peak-limiting, amplitude compression, non-linear gain, frequency shifting, and many types of noise reduction algorithms. The application and adjustment of these parameters can have a significant effect on music perception and appreciation [5]. For example, pitch may be altered by the use of frequency shifting, and loudness cues may be altered by the use of non-linear gain functions. Despite these possible alterations, the signal processing algorithms in HAs generally do not totally abolish the spectral information which encodes streaming cues such as pitch.

To our knowledge there are no published studies investigating stream segregation using non-speech stimuli in hearing-impaired listeners while they are using their hearing aids. However there have been several investigations, in un-aided listeners, of the effect of hearing loss itself on the ability to separate auditory streams. In a melody recognition task conducted with hearing-impaired (HI) and normally-hearing (NH) listeners, de Laat and Plomp [13] found that compared to NH listeners, HI listeners required a much larger pitch separation between melody and masker notes in order to recognise the melody. In sequential streaming tasks, such as van Noorden's previously described ABA-ABA task, there is a wider variety of results. For instance, Rose and Moore [14] found that only a proportion of individual HI listeners needed a large δF compared to NH listeners in order to segregate ABA-ABA pure tones. This was later shown to be unrelated to the amount of hearing loss, and loss of frequency selectivity in the HI listeners [15]. On the other hand, Grimault *et al.* [16] showed that with harmonic complex tones, elderly hearing-impaired participants showed significantly lower stream segregation scores than young normally-hearing participants. Hearing loss itself thus does not always have a detrimental effect on the ability to segregate auditory streams based only on pitch cues, but it is as yet unknown how the use of a hearing aid affects this ability. Although HAs may cause perceptual alterations in the types of cues used for auditory streaming, these altered cues may still be usable for the task of segregating sound sources.

Most contemporary CIs operate by converting the amplitude envelopes of the outputs of a series of between 16 and 22 bandpass filters into current levels, which are sent to an electrode array within the cochlea. As only the amplitude envelopes are encoded, most spectral detail is discarded, and the sensation of pitch is mainly elicited by manipulating the region of the cochlea stimulated by different electrodes (although temporal cues within bands can also contribute to pitch sensations below approximately 300 Hz [17], [18]. Streaming cues that depend on spectral detail, such as pitch and timbre, are thus much reduced in CI users.

Previous research investigating stream segregation using interleaved stimuli in CI users has generally reported that streaming in typical A-B-A tasks using pitch cues is difficult [19], [20] if not impossible [21], [22]. Given that place of stimulation (ie the location of the electrode stimulated) is thought to be a major contributor to pitch sensation in CI users [23], these studies have used stimuli limited to single electrodes, either via direct stimulation of discrete electrodes for the A and B tones [19], or by using pure tones with frequencies matched to the centre frequency of each band in the sound processor [21]. However, in streaming tasks, where any perceptual difference between streams can act as a cue to assist in segregation, the use of complex acoustic stimuli, which may introduce cues other than pitch, may in fact increase the ability to segregate streams, despite a possibly less accurate perception of relative pitch.

In addition to bottom-up or acoustic cues, steam segregation can be affected by top-down factors, such as diverting attention to competing auditory [24] or visual [25] tasks, or dynamic auditory contexts [26], [27]. The manipulation of concurrent audio-visual stimuli has also been shown by Rahne *et al.* [28] to influence stream segregation. In this study, the frequency separation and rate of a sequence of high and low tones was chosen so that the perception could be either one or two streams. A visual stimulus, arranged to reinforce either the one- or two-stream interpretation, was found to produce a bias towards the corresponding auditory perception, and also influenced mismatch-negativity brain responses to occasional deviants in the auditory sequence. In a musical streaming experiment, Marozeau *et al.* [29] showed that visual cues could reduce the difficulty of segregating a simple four-note melody from a background of random distracter notes. In that experiment, the four melody notes were displayed on a simple musical stave, and lit up in red as each melody note played. This visual cue reduced subjective difficulty ratings by approximately 14% for normally-hearing listeners with no training or familiarity with reading music. Together, these results show that visual cues can influence the perceptual organisation of auditory streams in normally hearing listeners.

### Visual effects in listeners with impaired hearing

It has long been acknowledged that visual cues assist the hearing-impaired with the understanding of speech. Sumby and Pollack [30] showed that when observers with normal hearing could both see and hear a speaker, speech intelligibility improved to a level equivalent to a 15 dB increase in the signal to noise ratio, and similar improvements are found in hearing-impaired listeners [31]. More recently, Devergie *et al.* [32] have shown that phonetically congruent video of lip movements presented during an A-B-A task using French vowel sounds increased obligatory streaming, suggesting that part of the gain provided by visual cues in speech understanding is due to visual enhancement of obligatory auditory streaming. Although there are significant changes in the visual system that are associated with hearing loss [33], these gains do not appear to be due to improvements in basic visual abilities [34]. Rather than changes in uni-sensory processing abilities, the gains found may be due to the improved ability to *integrate* auditory and visual information.

There are few studies investigating multisensory integration in listeners with impaired hearing, and the results are mixed. Cochlear implant users have been shown to be better than normally-hearing listeners at integrating visual information with degraded speech signals, even after accounting for increases in lip-reading proficiency [35]. In an investigation of the McGurk effect [36], Tremblay *et al.* [37] showed that CI users performed similarly to NH listeners, and a strong correlation was found between CI listening proficiency and the ability to integrate auditory and visual information. However, it has also been shown that in a passive listening task using synthetic syllables, CI users do not show the same decrease in latency and amplitude of P1 and N1 event-related potentials that normally indicate increased multisensory integration in NH listeners [38].

Nevertheless, in normally-hearing listeners, visual information has been shown to influence stream segregation [28], and can reduce the subjective difficulty of extracting simple melodies from random background notes [29]. If visual information can improve stream segregation in a musical context, people with hearing impairments may also be better able to take advantage of this information. The provision of an appropriate visual cue may thus improve the appreciation of music for users of cochlear implants and hearing aids.

The present study used the musical streaming paradigm from Marozeau *et al.* [29] to determine whether hearing-impaired listeners using HAs or CIs show the same visually-induced reduction in subjective difficulty in a stream segregation task as NH listeners. Listeners with CIs, HAs, and NH listeners were asked to continuously rate the subjective difficulty of extracting a simple, repeating, 4-note melody from a background of interleaved pseudo-random distracter notes. The distracter notes were chosen from an octave-wide pool, and the pitch range of this pool gradually varied throughout each block. Thus the main acoustic cue available to separate the melody and distracter notes was pitch. This task has been used in previous studies of auditory streaming in listeners with normal hearing, and has been shown to correlate well with an objective measure of stream segregation based on a detection task [29]. As HA listeners may have more pitch cues available than CI users, we hypothesised that HA users would rate the task as less difficult than CI users. Given previous evidence suggesting that hearing impairment itself does not affect streaming ability based on pitch cues, we further speculated that some HA users may not find the task any more difficult than NH listeners. It has been shown previously that for NH listeners, the task of extracting the four-note melody can be rendered less difficult if a visual cue reflecting the melody notes pitches is available [29]. Given previous research suggesting that HI listeners may integrate visual cues with degraded auditory information just as well, or even more effectively than NH listeners [31], [35], [37], we also hypothesised that both HA and CI listeners would report less difficulty with this streaming task when visual cues were available. The findings have implications for the design of devices that may help the hearing impaired appreciate and enjoy music.

## Methods

### Participants

#### Ethics Statement

The experimental protocol conforms to The Code of Ethics of the World Medical Association (Declaration of Helsinki), was conducted at the Bionics Institute in Melbourne, Australia, and was approved by the Human Research Ethics Committee of the Royal Victorian Eye and Ear Hospital (Project 09-880H). Written, informed consent was obtained from all participants prior to participation in the study.

A total of forty-two participants were recruited. Twenty participants with normal hearing (10 female) were recruited through a combination of social networks and advertisements on the Bionics Institute website. All these participants had been part of other studies involving streaming rating judgments, and were not musically trained based on a self-report musical aptitude scale [29]. All these participants reported normal hearing, and all participants reported normal or corrected to normal colour vision.

Eleven CI users (5 female) were recruited from the Cochlear Implant Clinic at the Royal Victorian Eye and Ear Hospital in Melbourne, Australia. All participants had profound post-lingual sensorineural hearing loss of hereditary or unknown origin in both ears. All participants had a minimum of three years experience with their CI, and the standard everyday program was selected on their sound processor. No adjustments were made to program, gain, or sensitivity settings during the testing session. If a hearing aid was prescribed for the contralateral ear, it was switched off and replaced by an ear plug. One participant, CI_11, had bilateral cochlear implants, both of these were switched on and operating as usual. Age, sex, and cochlear implant details are shown in Table 1.

**Table 1:** Cochlear implant participant details.

ID	Age	Sex	Implant	Sound Processor	Strategy	Rate per channel	Side	Age at Implant - L	Age at implant - R
CI_01	66	male	Nucleus CI24R CA	ESPrit 3G	ACE	720	right	NA	62
CI_02	58	female	Nucleus CI24R CA	Freedom SP	ACE	900	right	NA	53
CI_03	58	male	Nucleus CI22M	Spear R3	SPEAK	250	left	45	NA
CI_04	75	female	Nucleus CI24M	Esprit 3G	ACE	900	right	NA	67
CI_05	87	female	Nucleus CI24M	Esprit 3G	SPEAK	250	right	NA	78
CI_06	72	male	Nucleus CI24RE CA	Freedom SP	ACE	900	left	69	NA
CI_07	69	male	Nucleus CI24RE CA	Freedom SP	ACE	900	right	NA	66
CI_08	64	male	Nucleus CI24M	ESPrit	SPEAK	250	right	60	NA
CI_09	57	male	Nucleus CI24M	SPrint	ACE	900	left	NA	43
CI_10	68	female	Nucleus CI24R CS	Freedom SP	ACE	900	both	61	NA
CI_11	61	female	L: Nucleus CI24R CS + R: Nucleus CI24RE CA	Freedom SP	ACE	900	left	55	61

**Footnote**: All implants are 22-channel Cochlear Nucleus™ implants. CA: Contour Advanced. CS: Contour Straight. ACE: Advanced Combination Encoder. SPEAK: Spectral Peak.

**Figure 1:**
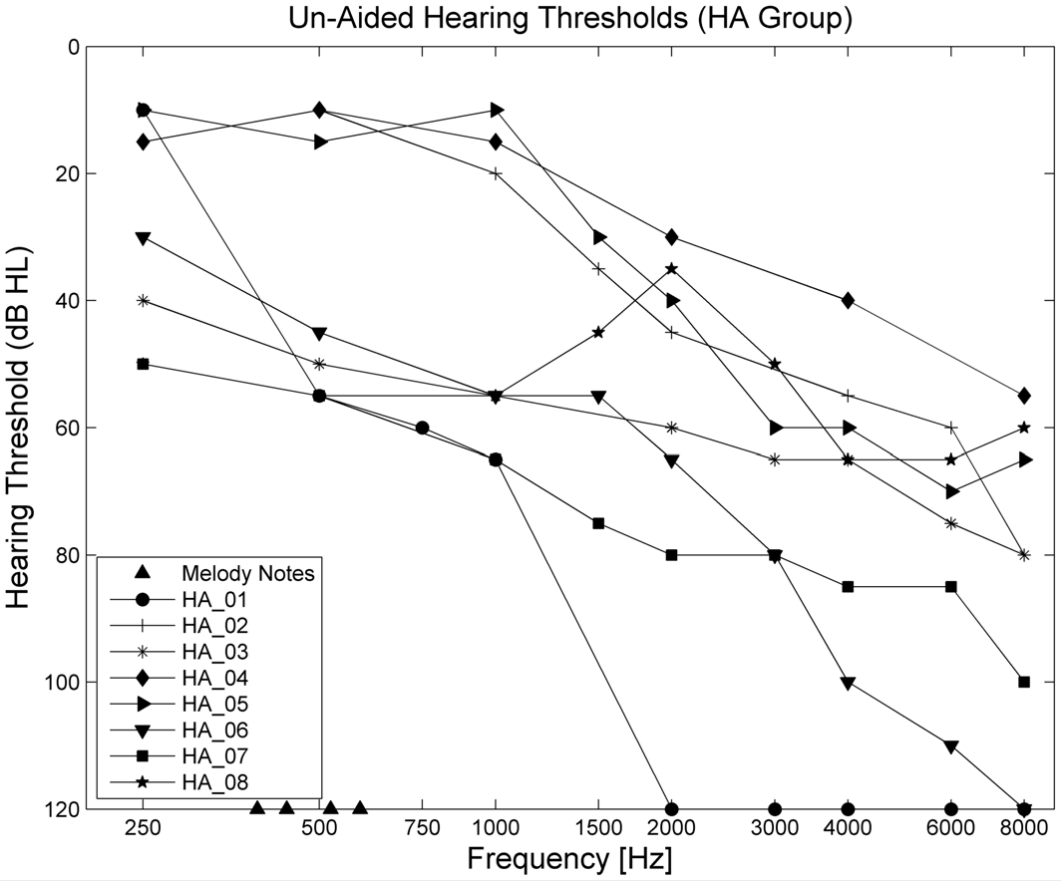
Audiograms for HA group. Best-ear un-aided audiogram results from the HA group. Hearing thresholds are given in dB HL. The fundamental frequency of the melody notes is shown by the black triangles on the x-axis.

Ten HA users (1 female) were recruited from local hearing aid clinics. All HA users had bilateral aids, and both were switched on, using their normal everyday programs. All HA users had a minimum of 2 years experience with their HAs. Age, sex and HA details are shown in Table 2. Two HA users were excluded from the study as they did not understand the task instructions. Figure 1 shows the unaided audiograms of the HA listeners. The fundamental frequency of each melody note is indicated with black triangles on the x-axis. All HA users had un-aided pure-tone hearing thresholds of 55 dB HL or less at 500 Hz. The stimuli were presented at 65 dB SPL, and HAs were active during the experiment. When the melody notes were played alone, with no interleaved distracter notes, all participants in every group reported that they were able to hear the melody notes, and that they could detect a four-note, repeating pattern.

**Table 2:** Hearing aid participant details.

ID	Age	Sex	HA type	Side	Age at Fitting - Left	Age at fitting - Right
HA_01	52	male	Phonak Supero	both	22	22
HA_02	71	male	Sonic Velocity Mini	both	69	69
HA_03	84	male	Phonak Naida IX	both	74	78
HA_04	65	male	Audéo YES III	both	64	64
HA_05	70	male	Siemens Destiny	both	68	68
HA_06	87	male	Phonak Supero 412	both	60	60
HA_07	86	male	Phonak Naida IX	both	75	78
HA_09	72	female	Siemens Destiny	both	70	70

### Subjective streaming difficulty task

Recent work has emphasised the development of objective, performance-based measures of auditory streaming [39], [40]. These measures involve a listener being asked to carry out a task in which performance is either improved or degraded by stream segregation. For instance, it is more difficult to detect changes in the timing of successive stimuli when alternating stimuli fall into separate streams compared to when they are integrated. Thus, the experimenter can infer whether the percept is integrated or segregated based on the performance of participants when they are asked to detect timing irregularities rather than make subjective reports on stream segregation itself. These types of tasks have the advantage of being less affected by the different response biases of individual participants, and are particularly advantageous when used with neuro-imaging techniques, when it is important that the stimuli in various conditions remain as constant as possible, or in animal studies, where the concept of streaming is impossible to explain [40]. In the current study, however, these issues were not a concern, and a more direct, subjective report approach was preferred. As the aim was to address stream segregation in music, the task was also designed to be musically valid where possible.

Performance-based tasks such as those mentioned above generally measure the temporal coherence boundary (TCB – although see Experiment 3 of Micheyl & Oxenham [40] for a recent exception) – the point at which the difference between streams is so large that *obligatory streaming* occurs [8]. In these tasks, performance on a detection task starts to decline when the difference between the two streams is greater than the TCB, where listeners always hear two separate streams. However, in the context of the current study, the aim was to determine the effect of visual cues on the *minimum* difference that allowed participants to *start* to segregate the streams. Thus, the task in the current experiment was designed to measure the fission boundary (FB) [8].

A musical streaming paradigm was thus employed where participants provided a direct, subjective report of the difficulty of segregating a simple, four-note *target melody* from a background of interleaved random *distracter notes*. This task has been used previously to assess the effect of visual cues on auditory stream segregation in normally-hearing musicians and non-musicians [29]. The current study extends these results to HA and CI users. In this paradigm, the distracter notes gradually changed in pitch, from completely overlapping the target melody, in which case it was difficult or impossible to segregate the melody, to completely separated from the melody, in which case it was very easy to segregate the target melody. While the target melody was constantly repeated along with the interleaved distracter notes, the participant was asked to continuously rate the subjective difficulty of segregating the target melody, using a variable slider marked from ‘easy’ to ‘impossible.’

In previous work using this task [29], the subjective difficulty ratings from normally-hearing participants were compared with results from an objective, performance-based detection task using an otherwise similar experimental paradigm in the same participants. In the streaming detection task, the target melody was occasionally altered by inverting the order of two of the four notes, and participants were asked to detect the occasional inversions of the melody rather than rate the subjective difficulty. As the interleaved distracter notes began to overlap the target melody, the melody became difficult to segregate, and the occasional melody inversions became more difficult to detect. When the average difficulty ratings from the difficulty rating task were compared with the miss rate in the detection task, it was found that there was no systematic difference in how individuals responded in each task – those that reported low difficulty ratings in the subjective task also reported low miss rates (high accuracy) in the objective task, and vice-versa.

As the current study included visual cues that represented the pitches of the target melody, it would be difficult to introduce an objective detection task such as that employed as a control in Marozeau *et al.* [29]. If the visual display were kept consistent with the target melody (including the deviant sequences), participants could perform the task purely visually. On the other hand, if the visual display continued to present the un-altered target melody while deviant sequences were playing, the mis-match between the auditory and visual stimuli might confuse participants, and make the results difficult to interpret. Finally, the current study employed a subtraction method, where ratings from each participant in the No-Vision blocks were subtracted from those in the Vision blocks. Any within-subject response bias would thus be cancelled. For these reasons, only a subjective streaming difficulty rating task was performed in the current study.

### Stimuli and apparatus

The target melody and distracter notes were constructed using Matlab 7.5 and presented using a standard PC (Dell Optiplex 960: Dell, Texas, USA) running MAX/MSP 5 software (Cycling 74, San Francisco, USA) through an M-AUDIO (AVID Technology, California, USA) 48-kHz 24-bit Firewire sound card. Each note consisted of a 180 ms complex tone with 10 harmonics. Each successive harmonic was attenuated by 3 dB, and each note included a 30 ms raised-cosine onset and 10 ms offset. The notes were played from a loudspeaker (Genelec 8020B, Iisalmi, Finland) positioned on a stand at the listener's ear height, 1 m from the listener's head. Each note was equalised in loudness to 65 phons according to a loudness model [41].

**Figure 2:**
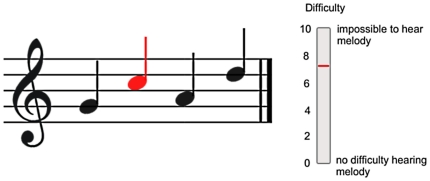
Visual display. The simple 4-note melody (G, C, A, D, midinotes 67, 72, 69, 74) depicted on the stave used as the visual display. Each melody note turned red as it played. The scale to the right repeated the participants' response in real time, so they did not have to look away from the screen to gauge their response.

The visual cue was also generated with MAX/MSP 5. It consisted of a musical stave with the 4-note target melody depicted in standard musical notation (see Figure 2). Each note in the visual cue turned red as the appropriate melody note played. In this way, the visual cue depicted the shape of the whole melody, as well as the current note playing. The synchronisation of the auditory-visual cue was measured by recording the output of a light-sensitive diode simultaneously with the audio output to a 2-track audio file sampled at 44.1 KHz. By comparing the onset times of the signals from the light-sensitive diode and the auditory stimulus, it was possible to calculate the delay between the two. The visual cue led the auditory stimulus consistently by 36 ms. To ensure participants did not have to look down at the response slider during the experiment, a visual depiction of the response slider was shown on the screen immediately to the right of the stave. The current position value of the slider was updated in real time and shown in red. Video examples of the visual and auditory stimuli can be found in Supporting Information files S1 and files S2 online.

**Figure 3:**
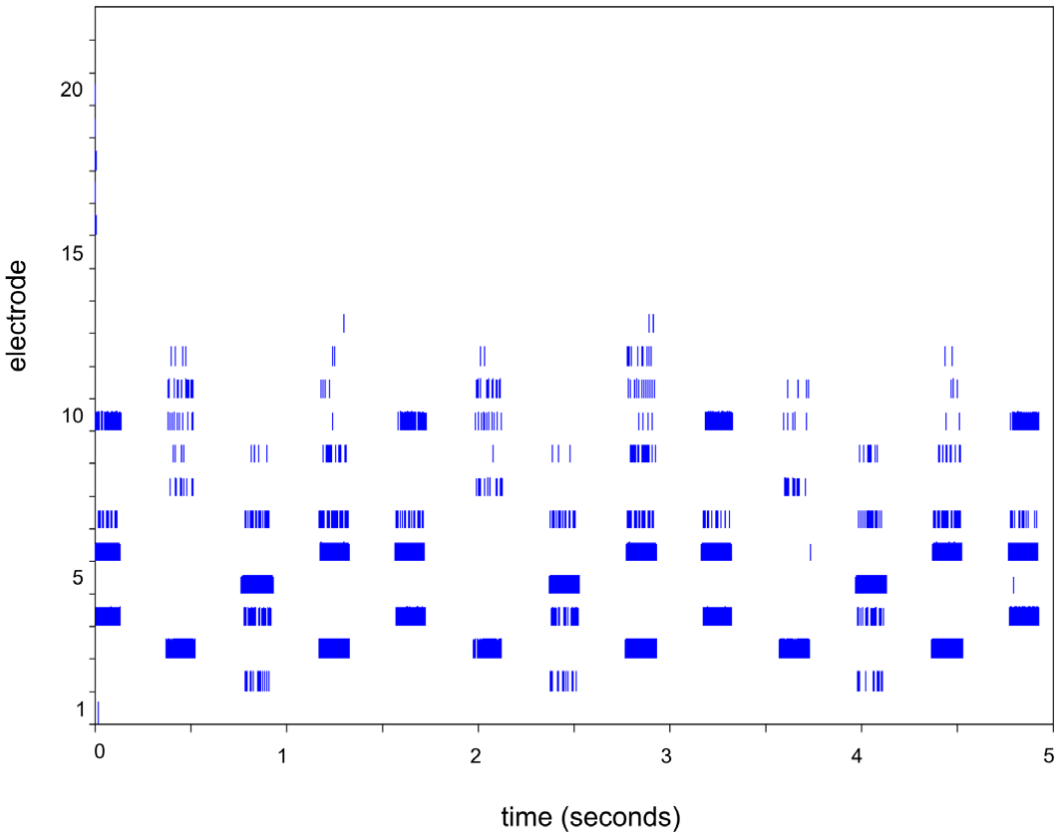
Electrodogram for melody notes. An “electrodogram” showing the stimulation across electrodes (on the y-axis) over time, as the 4-note melody is repeated three times. The first note of the melody starts at time 0. The electrodogram was generated by RFStatistics software (Hearing CRC, Melbourne).

The pattern of electrical stimulation for the 4-note melody, delivered acoustically to the participants own sound processor, was recorded for each CI participant using RFstatistics (software developed by the Cooperative Research Centre for Cochlear Implant and Hearing Aid Innovation, Melbourne, Australia). An example from one participant is shown in Figure 3.

### Procedure

In the subjective difficulty rating task, the four-note target melody spanned 8 semitones, while the distracter note pitches were randomly chosen from a 12-semitone wide range of notes. The target melody pitches (see Figure 2) were G, C, A, and D above middle C (midinotes 67, 72, 69, and 74 respectively). As the experiment was designed to be performed by listeners with hearing impairment as well as the normally-hearing, the melody was composed of intervals large enough to be perceived by participants with poor pitch discrimination (as is often the case in cochlear implant listeners) while being small enough for the sequence to be grouped into a single stream (instead of two interleaved streams composed of the two lowest notes and two highest notes). For convenience, note pitches are referred to throughout using standard midinote values–middle C was designated ‘midinote 60,’ with each integer corresponding to a semitone change in pitch.

**Figure 4:**
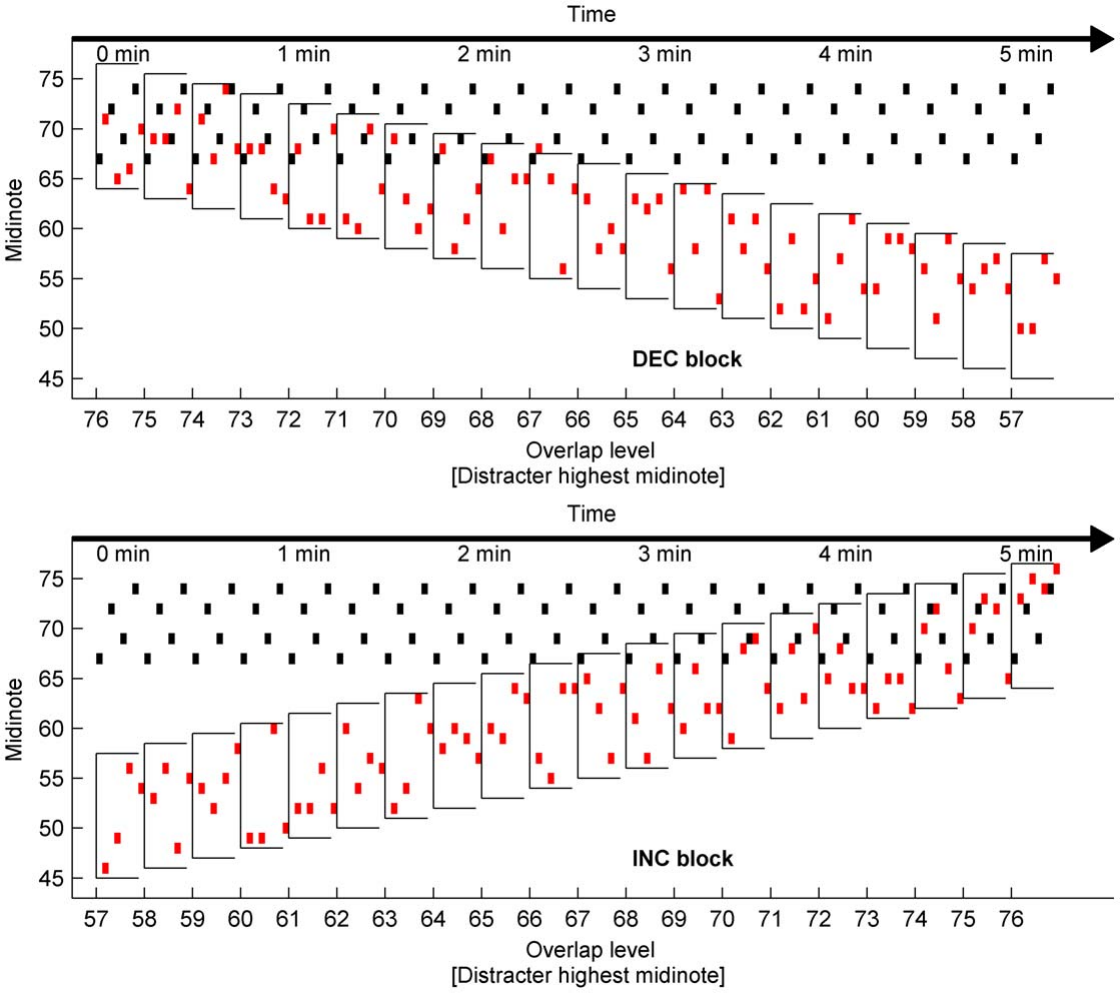
Task design. Decreasing (DEC: upper panel) and increasing (INC: lower panel) blocks are shown. Melody notes (black/dark dots) play continuously. Distracter notes (red/light dots) are interleaved with the melody notes, and are selected from a range of 12 consecutive midinotes (an octave). The distracter note range is increased or decreased by one midinote per level, for 20 levels. Within each level, the melody is repeated 20 times (a single presentation is shown here).

Conditions with and without visual cues (see Figure 2 and Supporting Information Files S1 and S2 for examples) were run. In each of the Visual and Non-Visual conditions, four counterbalanced blocks were run for each participant: in decreasing blocks (DEC – upper panel of Figure 4), where the pitch range of the distracter gradually decreased, the melody notes started completely overlapped by the distracters, and ended with a separation of 11 midinotes (from the highest possible distracter note to the lowest melody note). The range of possible distracter notes decreased by 1 semitone in 20 pitch separation levels. The unqualified term ‘level’ will be used throughout to refer to experimental levels. The terms ‘loudness level’ or ‘intensity level’ will be used to refer to the sensation of loudness or the acoustic parameter associated with the sensation of loudness, respectively. Within each level, the melody was repeated 10 times (lasting 16 seconds). With decreasing overlap, the task became gradually easier. In increasing blocks (INC) the order was reversed and the experiment became gradually more difficult. The INC and DEC blocks were repeated twice each.

The participants were asked to rate the difficulty of perceiving the four-note melody continuously throughout each block using a variable slider on a midi controller (EDIROL U33). The slider was labeled from 0 (no difficulty hearing melody) to 10 (impossible to hear melody). The slider was initialized at the 0 position for INC blocks and at the 10 position for DEC blocks. Participants were instructed to move the slider to the “10” position if the melody was impossible to perceive and to the “0” position if the melody could be easily perceived, and to start moving the slider to reflect their perceived difficulty level as soon as the block began. The position of the slider (encoded with 128 possible difficulty levels) was recorded for each melody repetition and stored for later analysis.

The level of the distractor was changed gradually and monotonically, rather than in a randomised fashion, in order to minimise the streaming build-up effect [7]. When a listener is presented with a new sequence of two streams they are initially perceived as fused. Depending on the differences between streams, the perception of separate stream then ‘builds up’ gradually. This effect can be reset by a variation in the differences between the streams. Therefore, the incremental variation of the distractor used in the current study was designed to prevent this re-setting of the streaming perception.

## Results

**Figure 5:**
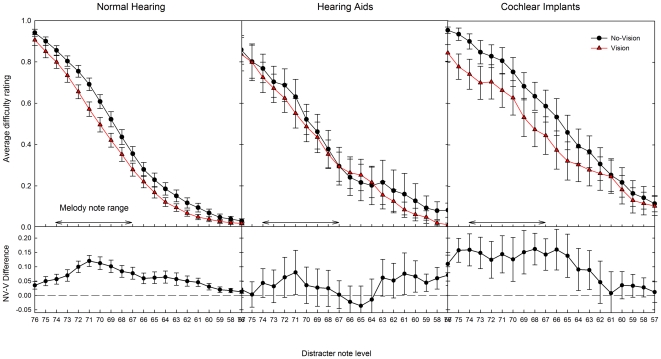
Difficulty ratings across distracter note levels. Top panel: Difficulty ratings (+/- SEM), averaged across INC and DEC blocks, as a function of distracter note level, with visual cues provided (red triangles) and with no visual cues (black squares). Bottom panel: the reduction in difficulty provided by the visual cue.

Figure 5 (upper panel) shows the difficulty ratings, averaged across repeat and direction, as a function of distracter note level, for Vision and No-Vision blocks, and for NH, HA and CI listeners. When the distracter note level was high (when the distracter notes overlapped the melody), all participants rated the task as very difficult or impossible. As the distracter note level decreased, average difficulty levels decreased in a monotonic fashion until the lowest distracter note level, when the distracter notes were maximally separated from the melody notes. At this point, most participants reported that the melody notes were very easy to separate from the distracters.

**Figure 6:**
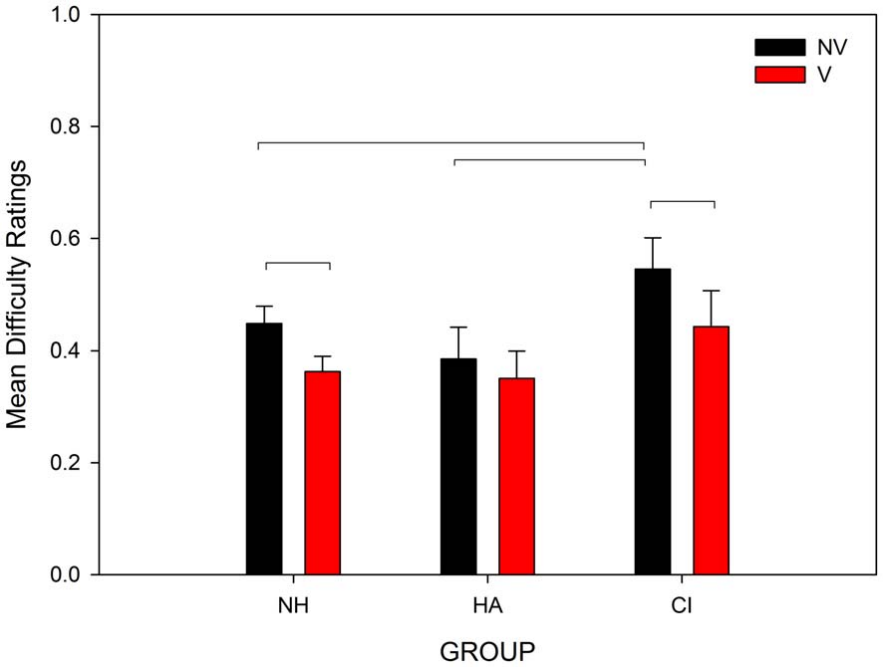
Mean difficulty ratings. Mean difficulty ratings across all distracter separation levels. Significant differences (Tukey HSD test) between groups and conditions are indicated with horizontal bars.

To quantify the effect of the visual cues on difficulty ratings, the difference between Vision and No-Vision blocks was calculated. This difference, corresponding to the reduction in difficulty provided by the visual cue, is shown as a function of distracter note level in Figure 5 (lower panel). In normally hearing listeners the maximum reduction in difficulty is around 12% of the maximum range. This figure is comparable to our previous study in NH non-musicians of 14% [29]. Hearing aid users do not appear to gain as much benefit from the visual cue, although the effect is still mostly positive. In CI listeners, the reduction is higher than in NH listeners, although with more variability evident between participants. For NH and CI listeners, the visual cue appears to provide the maximum benefit when the distracter notes are overlapping the melody, when the task was fairly difficult. The mean difficulty ratings across all levels are shown in Figure 6. With no visual cues present, CI users reported higher difficulty ratings than HA users and NH listeners. However, when visual cues were present, difficulty ratings from both NH listeners and CI users were lower, and all three groups reported similar mean difficulty ratings.

### Statistical analysis

In order to assess overall effects of the distracter separation level, the visual cue and direction of the distracter (INC vs DEC blocks), difficulty ratings were averaged across the two repeats for each condition, and submitted to a repeated measures mixed GLM, with a continuous within-subjects factor of Level (20 distracter note separation levels), categorical within-subjects factors of Vision (Vision, No-Vision), and Direction (INC and DEC blocks), and a between-subjects factor of Group (NH, HA, CI). The rating data were expected to follow a psychometric function with the level that was modelled as: rating  =  1/(1+exp(a*level+b)). Therefore the rating scores were transformed as: rating'  =  log(1/rating -1) in order to have a linear relationship between the rating and the level. Results were bounded to .01 and .99 prior to transformation in order to avoid infinite points. There was a small and borderline significant main effect of Direction, *F*(1,35)  =  4.3, *p* = .05, *r ^2^* = .004, with difficulty ratings in INC blocks generally lower than in DEC blocks. There were no significant interactions of Direction with either Level or Vision. As this effect had only borderline statistical significance, and was not important for interpreting the results, it was collapsed and the analysis was re-run The main factor Group was highly significant, *F*(1,35) = 25.7, *p*<.0001, *r^2^* = .021, as well as the main factor Level, *F*(1,35) = 656.7, *p*<.0001, *r^2^* = .93. There was a significant Group x Level x Vision three-way interaction, *F*(2,35) = 4.0, *p* = .03, *r* ^2^ = .002. The interaction was decomposed using Tukey HSD tests. Means across all levels are plotted in Figure 6. In No-Vision blocks, the CI users rated the task as more difficult overall than both NH listeners (*p*<.001), and HA users (*p*<.001). There were significant differences between Vision and No-Vision blocks for the NH ( *p* = .007) and CI (*p* = .02) groups, but not for HA users.

## Discussion

Our previous research [29] has shown that normally hearing listeners are able to use simple visual cues depicting pitch to reduce the subjective difficulty of segregating a simple melody from interleaved random background notes. The current study employed the same paradigm to show that CI users can also use these basic visual cues to the same advantage. These results have significant implications for the design of future auditory-visual aids that may make music more enjoyable for CI users. HA users, however, did not gain any significant benefit from the visual cues.

The present study employed a musical stream segregation paradigm, where participants were asked to continuously rate the subjective difficulty of segregating a simple four-note melody from a background of interleaved random distracter notes. As the distracter notes increased in pitch towards the melody notes, difficulty ratings generally increased monotonically. This result replicates our previous study [29], and is in agreement with previous research examining the ability to segregate melodies from interleaved distracter notes [42]. In Dowling's studies, participants were required to name a familiar melody rather than rate the difficulty of extracting a repeating melody, however the results are similar – when the distracter notes completely overlapped the range of the melody notes, the participants in Dowling's experiments were generally unable to name the familiar melodies. As the interleaved distracter notes decreased in pitch, away from the range of melody notes, participants began to name the familiar melodies. A similar pattern was seen in the current study – participants were generally unable to segregate the melody while the distracter notes overlapped in pitch.

### Effect of visual cues

The main finding from the current study was that the presentation of a simple visual cue significantly reduced the subjective difficulty of segregating a simple 4-note melody from a background of interleaved random distracter notes in normal-hearing listeners and those using a cochlear implant, but the effect was weaker and more variable in hearing aid users.

There is now a large body of literature describing how congruent audio-visual stimuli of many different types are recognised faster and detected more accurately at near-threshold levels than either the visual or auditory stimulus alone [43]. Visual information has also been shown to influence stream segregation [28]. In Rahne *et al.* [28], a visual stimulus, consisting of boxes sized according to the frequency of each auditory stimulus, was designed to complement either a segregated or integrated perception of the auditory stream. A mismatch negativity response to occasional deviant patterns within one of the auditory streams was found only when the visual cue corresponded with the *segregated* auditory perception. The visual cue thus acted to resolve an ambiguous auditory sequence.

The effect of visual stimuli on auditory processing has also been described at low levels in the brain. It has been shown that visual cues presented at the same time as auditory stimuli can reduce reaction times [44], [45], improve the detection of low-intensity sounds [46], and increase the perceived loudness of low-intensity sounds [47]. It has also been shown that visual stimuli can improve the encoding of pitch and timbre in the auditory brainstem, particularly in musicians [48], [49], and increase the stimulus-related information content of neuronal firing patterns in the auditory cortex [50]. Of particular interest in relation to hearing-impairment research is the fact that the behavioural benefits of multisensory stimuli are strongest when one or more of the constituent stimuli alone elicits only a weak response [51].

When the visual cues were present, the grand mean difficulty rating for CI users was not significantly different to normal-hearing listeners without the benefit of the visual cue (Figure 6). Whether the visual effect on streaming is a result of improved encoding of acoustic features in the brainstem and cortex, or due to more top-down effects of the visual stimulus, is currently unknown, and a topic for further investigation. However, improvements in the representation of acoustic features in the brainstem and cortex may lead to more salient perceptual differences between sounds, and hence this mechanism could conceivably explain the effects of visual stimuli found in Rahne *et al.* [28] as well as in the current experiment.

### Hearing aid results

Hearing aid users in the current study reported overall difficulty ratings that were no higher than the in NH group. Indeed, although the difference was not statistically significant, the mean difficulty ratings in both the Visual and Non-Visual conditions were slightly lower than in the NH group (Figure 6). This was not unexpected given previous research showing that un-aided hearing loss itself does not always have a detrimental effect in pure-tone sequential streaming tasks using pitch cues [14], [15]. In the current study, however, the participants were using their hearing aids. Although the devices may have altered pitch cues through frequency-shifting algorithms, the altered cues were still sufficient for the participants using hearing aids to perform the streaming task as well as those with normal hearing. One possibility explaining the slight *reduction* in difficulty ratings for the HA users is that the complex harmonic stimuli may have introduced additional perceptual cues dependant on F0: as F0 increases, the higher harmonics in the stimuli may be relatively more attenuated in the HA group compared to the NH group, thus introducing a change in timbre. This cue could be used in addition to pitch to perform the streaming task [52]. A future experiment with HA users assessing the change in timbre with F0 in harmonic stimuli would be required to test this speculation.

Unlike the NH group and the CI users, HA users in the current study gained only a small and statistically insignificant benefit from the visual cues. The result cannot be explained by floor effects, as when the distracter notes were overlapping the melody, the HA group still reported high levels of difficulty in extracting the melody. Given previous research showing that hearing-impaired listeners gain benefit from visual cues in speech perception [31], this result was unexpected. However, a similar result was found in our previous study with musicians and non-musicians using the same task [29]. In this study, normal-hearing non-musicians showed a similar reduction in difficulty when visual cues were present. However, musically-trained participants, although they rated the task as generally less difficult, did not show the same reduction in difficulty when the visual cues were present, despite their years of training using the visual cue (as in the current study, it consisted of a musical stave). In Marozeau *et al.* [29], it was speculated that although highly-trained musicians were familiar with scores, the score may not provide the most salient visual cue when musicians are faced with difficult stream-segregation tasks. In such cases, as in an orchestra, the score may be either ignored, or combined with other visual cues, such as the conductor or other players movements, or purely auditory cues. The HA users in the current study may face a similar situation in their every-day lives. In informal post-experiment communications, many of the HA users in the current study reported that they found the task conceptually similar to many real-life situations, in that they were required to sort or classify sound sources. Having spent many years perfecting these auditory sorting skills, it is possible that the visual cue in the current experiment was simply not required. More formal research is required to address this intriguing possibility.

### Stream segregation in CI users

One intriguing result from the current study was that while CI users did report more difficulty extracting the melody than normally hearing listeners and hearing aid users (see Figure 5), their overall performance was better than previous research has suggested is possible [19], [20], [21], [22]. Previous research in this area has stressed the methodological importance of limiting the stimuli to single electrodes, either via direct stimulation of single electrodes [19] or by using pure tones with frequencies matched to the centre-frequency of each electrode [22]. Pure tones such as these are presumed to activate only a single electrode, and thus provide a more accurate perception of pitch. Whether activated by direct stimulation or by carefully-chosen pure tone acoustic stimuli, place of stimulation in the cochlea is known to convey at least some pitch information [53], although changing the stimulated electrode also elicits changes in percepts such as brightness and timbre [17], [54].

Melody recognition tasks, such as that conducted by Singh, Kong and Zeng [55], have shown that the ability of CI users to recognise melodies reduces as the harmonic complexity of the constituent tones increases. Harmonic sounds presented acoustically in free-field conditions are likely to stimulate one or more electrodes corresponding to the fundamental frequency, as well as a number of higher-frequency electrodes. These higher frequency electrodes may or may not correspond with the intended pattern of harmonics, likely providing an inaccurate perception of pitch [56], and thus reducing the ability to recognise melodies.

In the current study however, veridical recognition of the melody was not required. To maintain ecological validity, complex tones with ten harmonics (with a slope of 3dB/octave), were presented via loudspeaker in free-field conditions. The pattern across electrodes tended to be different for each note (see Figure 3 for an ‘electrodogram,’ showing melody notes only, from a single participant's sound processor), and might have led to increased perceptual differences, perhaps not only in pitch, between melody and distracter notes. Since the ability to segregate streams is thought to be based on all perceptual differences between sources, this may have led to an increase in the ability to segregate, independently of accurate pitch perception of each note.

### Task considerations

Subjective tasks have been used to assess various aspects of stream segregation in normally-hearing (for a review see Bregman, [6]) as well as hearing-impaired participants [19], [57]. In these studies, continuously-repeating patterns were used; however the tasks were to indicate whether one or two streams were perceived using a toggle switch (one-stream vs two-streams), rather than a continuous rating of the subjective *difficulty* of streaming as used in the current study. Reports of streaming difficulty have the advantage of being a direct measure of auditory streaming, but it is acknowledged that, by their nature, they are subjective reports, and will be influenced by individual participants' response biases. In order to address this issue, various objective detection tasks have been developed, where detection of a feature in a single stream is the dependant measure. For example, it is more difficult to detect alterations in the timing of sounds that occur within a stream than when they are in segregated streams [8], [58], [59], and it is more difficult to identify familiar melodies when interleaved distracter notes are overlapping compared to non-overlapping in pitch [42], [52]. Detection tasks such as these are more objective, have accuracy as the dependent measure, and are less susceptible to participant response bias, however they remain an indirect measure of stream segregation.

Several studies have compared performance on objective stream segregation tasks with a direct subjective measure. Using an objective temporal discrimination threshold measure of streaming in normally-hearing participants, Roberts, Glasberg & Moore [57] showed that differences between A and B streams in both the passband and component phase of complex tones with only high, unresolved harmonics could induce auditory streaming. When they repeated the experiment using a direct, subjective measure of streaming (the proportion of the time a toggle switch was flipped to a ‘two-streams’ rather than ‘one-stream’ position), they found the same pattern of results. Stainsby *et al.* [60] conducted similar experiments with elderly hearing-impaired participants, and found that although both passband and component phase increased stream segregation in the objective temporal discrimination task, only the component phase had a significant effect in the subjective streaming task. It should be noted, however, that the effect of the passband was small in both the objective and subjective tasks, and low numbers of participants (N = 5) and high subject variability may have contributed to the non-significant finding for passband in the subjective task. In a study of the effect of inter-aural time and level differences on stream segregation, Boehnke & Phillips [61] also found a strong correlation between their temporal discrimination and subjective streaming results. As previously described in the methods section, results from the task used in the current study have also been compared favourably with an objective measure of streaming [29].

The results from the present study show that the *subjective difficulty* of segregation was reduced for the NH and CI groups when visual cues were present. The analysis employed was a comparison between two conditions in the same subjects, thus individual response biases were likely cancelled. However, we acknowledge we cannot rule out the possibility that visually-induced changes in response criterion could explain the results. Nonetheless, in order to explain the differences between groups, any visually-induced change in response criterion would also have to act differently in each group. Further research using new paradigms will need to be undertaken in order to more fully explain these effects.

### Conclusion

The current study was undertaken to determine whether the provision of simple visual cues might improve the ability of hearing-impaired listeners to segregate a melody from background notes. It was shown that the provision of these cues could indeed reduce the difficulty of segregating the melody, but only in CI users, not HA users. Both hearing-impaired groups reported no more difficulty in segregating the melody than NH listeners when the visual cues were present. These results suggest that simple visual displays may be useful for cochlear implant users to improve their enjoyment of music. Further research is required to understand which acoustic cues to encode visually, the specific types of visual cues that are most useful, and whether improvements using these cues will generalise to other listening situations.

## Supporting Information

File S1 **Stimulus display (no-vision).** A video screenshot showing the stimulus display in no-vision blocks. The video is an example of an INC block with no visual stimulus, where the range of possible distracter notes gradually increases towards the melody, making it gradually more difficult to segregate the melody from distracter notes. The rate of change of the distracter note range has been increased to shorten the length of the demo video.
(MP4)

File S2 **Stimulus display (vision).** A video screenshot showing the stimulus display in no-vision blocks. The video is an example of an INC block with the visual stimulus, where the range of possible distracter notes gradually increases towards the melody, making it gradually more difficult to segregate the melody from distracter notes. The rate of change of the distracter note range has been increased to shorten the length of the demo video.
(MP4)
